# Comparative analysis of the clinical effect and safety of Laparoscopic Radical Cystectomy + Orthotopic Ileal Neobladder and Open Surgery

**DOI:** 10.12669/pjms.37.1.2273

**Published:** 2021

**Authors:** Yuan-hua Liu, Hai-tao Dai, Chang-mao Liu, Jiang Zheng

**Affiliations:** 1 Yuan-hua Liu, Department of Urology, Jingzhou First People’s Hospital, Jingzhou,434000, Hubei, P.R. China; 2 Hai-tao Dai, Department of Urology, Jingzhou First People’s Hospital, Jingzhou,434000, Hubei, P.R. China; 3 Chang-mao Liu, Department of Urology, Jingzhou First People’s Hospital, Jingzhou,434000, Hubei, P.R. China; 4 Jiang Zheng, Department of Urology, Jingzhou First People’s Hospital, Jingzhou,434000, Hubei, P.R. China

**Keywords:** Adverse reaction, Bladder tumor, Curative effect, Cystectomy, Orthotopic ileal neobladder

## Abstract

**Objectives:**

To explore the clinical effect and safety of laparoscopic radical cystectomy + orthotopic ileal neobladder and open surgery.

**Methods:**

The study was conducted at Jingzhou First People’s Hospital from January 2017 to July 2018. In this study 87 patients undergoing radical cystectomy + orthotopic ileal neobladder were chosen and classified into an observation group (48 cases) and a control group (39 cases) according to the surgical methods. The observation group underwent laparoscopic surgery, while the control group underwent open surgery. Perioperative period and prognostic conditions were compared in both groups.

**Results:**

The intraoperative bleeding amount obviously decreased. The recovery time of gastroenteric function and postoperative hospitalization time were significantly shortened. Postoperative pain was significantly alleviated. Compared with the control group, the observation group showed significant differences (P<0.05). The time, amount and difference in pelvic lymph node dissection in both groups were not significantly different (P>0.05). The differences in both groups in terms of the daytime/nighttime urinary continence rate, maximum urinary flow rate, internal bladder pressure, maximum bladder pressure during urination, internal urethral pressure, bladder capacity, and residual urine volume six months after the operation were not statistically significant (P>0.05). There was no significant difference in postoperative complications, including urinary fistula, bleeding, urinary tract infection, pulmonary infection, dysuria, lymphatic leakage, ureterostenosis, or relapse (P>0.05). The ileus incidence rate in the observation group was obviously lower than that in the control group, and the difference was statistically significant (P<0.05).

**Conclusion:**

Laparoscopic radical cystectomy + orthotopic ileal neobladder has the characteristics of limited trauma, a minimal amount of bleeding and a fast recovery. The functions of orthotopic neobladders are good, and the occurrence rate of postoperative complications is low. In addition, body immunity is protected. Hence, this procedure deserves to be promoted clinically.

## INTRODUCTION

Open radical cystectomy (ORC) has been the gold standard for treating muscular invasive bladder cancer^1^-^3^, and it is also one of the operations with the largest amount of trauma in the field of urinary surgery. Parra et al. reported on laparoscopic total cystectomy for the first time in 1992.^4^ With the development of laparoscopic instruments and surgical techniques in recent years, laparoscopic radical cystectomy (LRC) and orthotopic urinary diversion have been proven to be safe and feasible and have the advantages of limited trauma, a minimal amount of bleeding and a fast recovery.^5^,^6^ Orthotopic ileal neobladder (OIN), as a kind of urinary diversion after radical cystectomy, can closely mimic physiological urination in patients without the need for wearing urine collection bags, which greatly improves patients’ postoperative quality of life. Hence, it has become one of the preferred surgical methods for patients with invasive bladder cancer.^7^ OIN has been reported to account for 39.1%~74% of all urinary diversion surgeries with its application in multiple medical centers.^8^ Since the bladder is located deep in the human pelvic cavity, ORC has the disadvantages of a small operating space, a large amount of bleeding, and many complications.^9^,^10^ In addition, OIN has cumbersome operating procedures and long operation times. The feasibility of laparoscopic surgery has been disputed. In this study, the curative effects, neobladder functions, complications, and immunity indexes in patients were investigated after two surgeries to provide a reference for clinical treatment.

## METHODS

Eighty-seven patients who underwent radical cystectomy + orthotopic ileal neobladder in Jingzhou First People’s Hospital from January 2017 to July 2018 were chosen and classified into the observation group (48 cases) and control group (39 cases) according to the surgical methods. The observation group underwent laparoscopic surgery, while the control group underwent open surgery. Among the patients, there were 72 male patients and 15 female patients. The age was 40~70 years old, and the average age was (49.7±13.8) years old.

### Ethical approval

The study was approved by the Institutional Ethics Committee of Jingzhou First People’s Hospital at August 12 2019, and written informed consent was obtained from all participants

### Inclusion criteria

1. B ultrasound, CT, MRI, cystoscopy, and pathology verified the diagnosis of T2-T3, N0-x and M0 invasive bladder cancer.
2. Relapsed nonmuscular invasive bladder cancer.
3. Extensive papillary lesions due to ineffective conservative treatment.
4. Ineffective nonsurgical treatment or tumor recurrence after bladder-preserving surgery.

### Exclusion criteria

1. Follow-up visit could not be conducted.
2. Cardiopulmonary insufficiency, kidney disease or abnormal coagulation function.
3. Ureteral disease.

The differences in both groups in terms of sex, age, clinical manifestation, and neoplasm staging were not statistically significant (P>0.05), and the two groups were comparable, as shown in Table-I.

**Table-I:** Comparison of general data (X±S).

*Group*	*No.(n)*	*Sex(male/female)*	*Age(years)*	*Gross hematuria (n/%)*	*Neoplasm staging/No.*		

					T2N0M0	T3N0M0	T3N1M0
Observation group	48	41/7	65.9±5.3	27(56.3)	31	13	4
Control group	39	31/8	64.3±7.3	26(66.7)	27	8	4
t/ X^2^		0.191	1.762	0.590		0.551	
P		0.570	0.731	0.381		0.760	

### Surgical method

LRC for the observation group: the patient was placed in the lithotomy position. After general anesthesia, an incision was made at the inferior margin of the navel to create an artificial pneumoperitoneum and an operating hole. Noninvasive nippers were used to push away the intestinal canal, and the ureter was dissociated outside the bladder. The ureter was cut off, and the proper length was reserved. The ductus deferens was cut off, and the seminal vesicle was separated. The posterior wall of the prostate was dissociated to separate it from the rectum. The anterior wall of the bladder was dissociated, and the bladder was made full. Then, the endopelvic fascia was cut open, and the puboprostatic ligament was cut off. The complex of the dorsal vein of the penis was sutured and ligatured. The ligaments on the side of the bladder and prostate were cut off, and the nerve-vessel bundles were reserved to the greatest extent. The ligament on the posterior side of the bladder was cut off. The urethra was divided, and the prostate and bladder were excised. Bilateral lymph glands were dissected, and ileal bladder replacement was implemented. The ileum was pulled out of the body, and 15 cm of ileum with the pedicle 15 cm from the ileocecal junction was dissociated and cut off. The cut end of the ileum was sutured. The ileal cavity was washed, closed and embedded at the proximal end of the enteric cavity. A small opening was made on both sides. The cut end of the ureter was trimmed, and the stand tubes of the bilateral ureter were reserved. The opening of the allantois and the outer membrane of the muscular layer of the ureter were sutured. The mucous membrane of the distal-end opening was adjusted to the shape of a papilla. An oval incision was made in the lower right abdomen. The aponeurosis layer was cut open with a cross shape. The distal end of the ileum output end was fixed on the lower right part of the abdominal wall. The drainage tube was placed, and the incision was closed.

### ORC for the control group

the patient was placed in a supine position, and a 25~30 cm incision was made in the middle of the lower abdomen. The incision avoided the navel and was extended along the upper part of the abdomen. The ureter was marked at the bifurcation of the iliac vessels. The operation method was the same as that used for the observation group.

### Observation indicators

1. The following indicators in the perioperative period were compared in both groups: time of operation, amount of bleeding, recovery time of gastroenteric function, postoperative pain score, postoperative hospitalization time, and time and amount of pelvic lymph node dissection.
2. Daytime/nighttime urinary continence rates were recorded in both groups three months after the operation (0 or 1 safety pads were used every day). The urodynamic analyzer was used to examine urodynamic indexes in both groups six months after the operation: maximum urinary flow rate, bladder capacity, internal bladder pressure, bladder pressure in the filling period, maximum bladder pressure during urination, maximum urethral pressure, and residual urine volume.
3. Postoperative complications were recorded in both groups: ileus, urinary fistula, bleeding, urinary tract infection, pulmonary infection, dysuria, lymphatic leakage, ureterostenosis, and relapse.
4. Flow cytometry was used to detect immunity indicators one day before the operation and one day after the operation: CD3^+^, CD4^+^, CD8^+^ and CD4^+^/CD8^+^ T lymphocytes.

### Statistical method

SPSS 22.0 statistical software was employed for data analysis. The enumeration data are expressed as (%) and were tested with x^2^. The measurement data are expressed as x±s and were tested with t. Paired t tests were adopted for intragroup comparisons, and independent sample t tests were used for intergroup comparisons. P<0.05 indicates statistical significance.

## RESULTS

The patients in both groups underwent successful operations, and no deaths occurred in the perioperative period. No cases of transition to open surgery occurred in the observation group. The amount of bleeding in patients in the observation group obviously decreased. The recovery time of gastroenteric function and postoperative hospitalization time were significantly shortened. Postoperative pain was significantly alleviated. Compared with the control group, the observation group showed significant differences (P<0.05). The time, amount and difference in pelvic lymph node dissection in both groups were not significantly different (P>0.05), as shown in Table-II.

**Table-II:** Comparison of conditions in the perioperative period (X±S)

Group	No.	Time of operation(min)	Intraoperative bleeding amount (ml)	Recovery time of gastroenteric function (h)	Postoperative pain (VAS score)	Postoperative hospitalization time (d)	Time of lymph node dissection (min)	Amount of lymph node dissection (No.)
Observation group	48	315.2±34.6	347.5±19.4	86.2±13.5	4.66±1.2	18.4±4.7	109.6±24.6	24.9±8.7
Control group	39	327.1±47.3	739.3±31.5	110.3±12.8	6.83±2.3	22.9±7.4	119.3±25.4	23.2±9.8
t		1.291	64.055	9.070	0.296	2.197	-1.875	0.895
P		0.200	<0.001	<0.001	<0.001	0.017	0.064	0.373

The catheter was removed two weeks after the operation in both groups. Urinary fistula occurred in 3 patients, and the catheter was removed four weeks after the operation. A double J tube was removed 1 month after the operation in all patients. Most patients suffered from urinary continence, and urinary continence recovered within 1-2 months. The differences in neobladder function, daytime/nighttime urinary continence rate, maximum urinary flow rate, internal bladder pressure, maximum bladder pressure during urination, internal urethral pressure, bladder capacity, and residual urine volume six months after the operation between the two groups were not statistically significant (P>0.05). Intravenous urography results showed that the bladders in both groups was close to normal bladders, as shown in Table-III.

**Table-III:** Comparison of neobladder functions (X±S) (n/%)

Group	No.	Urinary continence rate (daytime/nighttime, %)	Maximum urinary flow rate (ml.s^-1^)	Internal bladder pressure (cmH_2_O)	Maximum bladder pressure (cmH_2_O)	Internal urethral pressure(cmH_2_O)	Bladder capacity (ml)	Residual urine volume(ml)
Observation group	48	93.8/83.3	13.6±1.6	18.9±7.3	47.2±3.4	59.7±3.8	304.1±41.2	35.2±2.1
Control. group	39	89.7/84.6	13.4±1.3	18.1±9.4	46.7±2.3	58.9±4.1	289.1±37.1	34.9±1.8
t/ X^2^		2.069	0.866	0.292	0.632	0.838	1.211	1.098
P		0.150	0.195	0.386	0.265	0.202	0.118	0.139

The differences in urinary fistula, bleeding, urinary tract infection, pulmonary infection, dysuria, lymphatic leakage, ureterostenosis, and relapse between the two groups were not statistically significant (P>0.05). The ileus occurrence rate in the observation group was obviously lower than that in the control group, and the difference was statistically significant (P<0.05), as shown in Table-IV.

**Table-IV:** Comparison of postoperative complications (n/%)

Group	No.	Ileus	Urinary fistula	Bleeding	Urinary tract infection	Pulmonary infection	Dysuria	Lymphatic leakage	Ureterostenosis	Relapse
Observation group	48	2(4.16)	1(2.08)	1(2.08)	1(2.08)	1(2.08)	1(2.38)	1(2.08)	0(0)	1(2.38)
Control group	39	8(20.51)	2(5.31)	2(5.31)	3(7.69)	2(5.31)	1(2.38)	2(5.31)	0(0)	1(2.38)
X^2^		4.160	0.851	0.851	0.521	0.851	0.000	0.851	-	0.000
P		0.042	0.590	0.590	0.321	0.590	1.000	0.590	-	1.000

The number of CD8^+^ T lymphocytes in observation group was lower than that in the control group, and the number of CD3^+^, CD4^+^ and CD4^+^/CD8^+^ T lymphocytes was higher than in the control group. The differences were statistically significant (P<0.05), as shown in Table-V.

**Table-V:** Immunity comparison (X±S).

*Group*	*No.*	*CD3^+^ (%)*		*CD4^+^ (%)*		*CD8^+^ (%)*		*CD4^+^/CD8^+^*	

		Preoperation	Postoperation	Preoperation	Postoperation	Preoperation	Postoperation	Preoperation	Postoperation
Observation group	48	61.71±6.51	50.34±7.80*	37.01±5.52	32.71±7.60*	23.55±3.01	28.56±2.40*	1.56±0.21	1.32±0.16*
Control group	39	61.80±4.71	34.76±7.21*	36.52±5.48	26.77±3.32*	23.68±2.81	39.98±4.20*	1.54±0.19	0.88±0.31*
t		-0.636	10.278	0.408	13.399	0.636	20.979	0.032	13.369
P		0.527	<0.001	0.684	<0.001	0.527	<0.001	0.974	<0.001

*Note:* comparison before and after the operation: P

* <0.05

## DISCUSSION

As the concept of minimally invasive treatment becomes increasingly relevant and as laparoscopic techniques continue to improve, laparoscopic surgery has become an important treatment for urinary bladder carcinoma.^11^ Some experts have proven that laparoscopic radical cystectomy and orthotopic ileal neobladder can achieve the established oncology standard of open surgery.^12^ In addition, this study found that the amount of bleeding in the LRC + OIN group decreased obviously compared with that in the ORC group. The recovery time of gastroenteric function and postoperative hospitalization time were significantly shortened. Postoperative pain was significantly alleviated.

The possible reasons for these findings are as follows:

1. The amplification effect of laparoscopy can help the operator better recognize tissues and blood vessels. With the clearer surgical field and more meticulous operation, the probability of tissue and vessel damage and other damage can be reduced.
2. Pneumoperitoneum pressure can, to some extent, lower venous hemorrhage.
3. Laparoscopic instruments such as an ultrasound knife, bipolar coagulation, Ligasure and biological clips can better handle dissociation and excision.^13^
4. Laparoscopic surgery only slightly interferes with the intestinal canal. The exposure time of the intestinal canal in the air is relatively short, which can reduce traction for gastrointestinal tract tissues.^14^

Minimally invasive surgery has minimal trauma, and the intraoperative operation is meticulous. After the operation, patients’ pain is light, and body immunity can recover quickly. Hence, patients can get out of bed early.

Urinary diversion after radical cystectomy can be conducted in many ways, but OIN has been considered a relatively effective surgery.^15^ Compared with those of other traditional urinary diversion surgeries, the physiological functions of orthotopic neobladders are very close to those of the original bladder.^16^ In this study, orthotopic neobladders in both groups six months after the operation displayed the features of high capacity, high compliance and low pressure, and the residual urine volume also gradually decreased with the extension of postoperative recovery time. Relevant research results showed that after orthotopic neobladder operation, daytime and nighttime urinary continence rates were 75%~95% and 50%~85%, respectively^17^, similar to the results of this study. Mateo E’s research indicated that transfusion rate, number and severity of complications are lower in laparoscopic cystectomy with ileal neobladder. No statistically significant impact on operative time and on hospital stay was observed. In patients undergone to laparoscopic approach, continence rate is lower but not statistically significant.^18^ In this study, the urinary continence rate, internal bladder pressure, internal urethral pressure, bladder capacity, and residual urine volume in the LRC + OIN group were slightly better than those in the open group. This is because lymph, nerves, vessels and the urethral sphincter in the pelvic floor can be clearly observed under the laparoscope, and the fine operation can reduce the damage to the neurovascular bundle and urethral sphincter, contributing to postoperative early urinary continence.

LRC + OIN has tedious operating procedures and a long operation duration, and the complication rate in the perioperative period can reach 28~64%.^19^ The main complications include ileus, uracratia, urinary fistula, urinary tract infection, pulmonary infection, and incision infection. Among them, ileus is the most common complication of LRC + OIN.^20^ In this study, the ileus incidence rate in the laparoscope group was obviously lower than that in the open group, which is related to the small interference damage to the intestinal canal during laparoscopic surgery.^14^ The occurrence rate of complications of laparoscopic surgery was slightly lower than that of open surgery, indicating that with the progression of laparoscopic technology, LRC + OIN may become a highly safe surgery. It has been reported that the operation can cause temporary immunity inhibition in the body, where the changes in T lymphocytes are most obvious: decreases in CD3+, CD4+ and CD4+/CD8+ decrease and increases in CD8+.^21^,^22^ This indicates that laparoscopic surgery can protect the immunologic mechanism of the body and significantly reduce postoperative infection and other complications.

### Limitations of the study

No prospective, randomized, controlled study was used in this study. It was only a retrospective study. In the next step, prospective, randomized, controlled studies are needed to further evaluate the advantages and disadvantages of the two procedures.

## CONCLUSION

LRC + OIN has the characteristics of limited trauma, a minimal amount of bleeding, a fast recovery, good neobladder function and a low occurrence rate of postoperative complications. In addition, a short-term curative effect has been affirmed. However, this study lacks observations with a large sample size and long-term follow-up visits, and the long-term curative effect needs to be further verified.

### Authors’ Contributions

**YHL and JZ** designed this study and prepared this manuscript, and are responsible and accountable for the accuracy or integrity of the work.

**HTD** collected and analyzed clinical data.

**CML** significantly revised this manuscript.

**JZ** is responsible and accountable for the accuracy or integrity of the work

## References

[1] Nguyen DP, Al Hussein Al Awamlh B, Wu X, O'Malley P, Inoyatov IM, Ayangbesan A (2015). Recurrence patterns after open and robot-assisted radical cystectomy for bladder cancer. Eur Urol.

[2] Patel R, Szymaniak J, Radadia K, Faiena I, Lasser M (2015). Controversies in Robotics:Open Versus Robotic Radical Cystectomy. Clin Genitourin Cancer.

[3] Kiss B, Burkhard FC, Thalmann GN (2016). Open radical cystectomy:still the gold standard for muscle invasive bladder cancer. World J Urol.

[4] Parra RO, Andrus CH, Jones JP, Boullier JA (1992). Laparoscopic cystectomy:initial report on a new treatment for the retained bladder. J Urol.

[5] Porpiglia F, Renard J, Billia M, Scoffone C, Cracco C, Terrone C (2007). Open versus laparoscopy-assisted radical cystectomy:results of a prospective study. J Endourol.

[6] Pruthi RS, Wallen EM (2008). Robotic-assisted laparoscopic radical cystoprostatectomy. Eur Urol.

[7] Wenyong L, Qingwen L, Sheng W, Beibei L, Jian L, Shuai Y (2017). Clinical efficacy analysis of laparoscopic radical cystectomy with orthotopic ileal neobladder in the treatment of invasive bladder cancer. J Mod Oncol.

[8] Minervini A, Serni S, Vittori G, Masieri L, Siena G, Lanciotti M (2014). Current indications and results of orthotopic ileal neobladder for bladder cancer. Expert Rev Anticancer Ther.

[9] Gakis G, Efstathiou J, Lerner SP, Cookson MS, Keegan KA, Guru KA (2013). ICUD-EAU International Consultation on Bladder Cancer 2012:Radical cystectomy and bladder preservation for muscle-invasive urothelial carcinoma of the bladder. Eur Urol.

[10] Parekh DJ, Messer J, Fitzgerald J, Ercole B, Svatek R (2013). Perioperative outcomes and oncologic efficacy from a pilot prospective randomized clinical trial of open versus robotic assisted radical cystectomy. J Urol.

[11] Weingarten TN, Taccolini AM, Ahle ST, Dietz KR, Dowd SS, Frank I (2016). Perioperative management and oncological outcomes following radical cystectomy for bladder cancer:a matched retrospective cohort study. Can J Anaesth.

[12] Huang J, Lin T, Liu H, Xu K, Zhang C, Jiang C (2010). Laparoscopic radical cystectomy with orthotopic ileal neobladder for bladder cancer:oncologic results of 171 cases with a median 3-year follow-up. Eur Urol.

[13] Aboumarzouk OM, Drewa T, Olejniczak P, Chlosta PL (2013). Laparoscopic versus open radical cystectomy for muscle-invasive bladder cancer:a single institute comparative analysis. Urol Int.

[14] Ginot R, Rouget B, Bensadoun H, Pasticier G, Bernhard JC, Capon G (2016). [Radical cystectomy with orthotopic neobladder replacement:Comparison of robotic assisted and open surgical route]. Prog Urol.

[15] Roghmann F, Becker A, Trinh QD, Djahangirian O, Tian Z, Meskawi M (2013). Updated assessment of neobladder utilization and morbidity according to urinary diversion after radicalcystectomy:A contemporary US-population-based cohort. Can Urol Assoc J.

[16] Herdiman O, Ong K, Johnson L, Lawrentschuk N (2013). Orthotopic bladder substitution (Neobladder):part II:postoperative complications management, and long-term follow-up. J Wound Ostomy Continence Nurs.

[17] Stenzl A, Cowan NC, De Santis M, Kuczyk MA, Merseburger AS, Ribal MJ (2012). Reply to Santhanam Sundar's letter to the editor Re:Arnulf Stenzl, Nigel C Cowan, Maria De Santis, et al. Treatment of muscle-invasive and metastatic bladder cancer:update of the EAU guidelines. Eur Urol 2011;59 1009-1018. Eur Urol.

[18] Mateo E, García-Tello A, Ramón de Fata F, Romero I, Núñez-Mora C, Angulo JC (2015). A comparative study between open and laparoscopic approach in radical cystectomy with orthotopic ileal neobladder. Actas Urol Esp.

[19] Hautmann RE, de Petriconi RC, Pfeiffer C, Volkmer BG (2012). Radical cystectomy for urothelial carcinoma of the bladder without neoadjuvant or adjuvant therapy:long-term results in 1100 patients. Eur Urol.

[20] Svatek RS, Fisher MB, Williams MB, Matin SF, Kamat AM, Grossman HB (2010). Age and body mass index are independent risk factors for the development of postoperative paralytic ileus after radical cystectomy. Urology.

[21] Faghih Z, Shobeiri SS, Ariafar A, Sarkarian M, Zeighami S, Nazari N (2016). CD8+T Lymphocyte Subsets in Bladder Tumor Draining Lymph Nodes. Iran J Immunol.

[22] Ma Z, Bao X, Gu J (2016). Effects of laparoscopic radical gastrectomy and the influence on immune function and inflammatory factors. Exp Ther Med.

